# Molecular signatures of premature aging in Major Depression and Substance Use Disorders

**DOI:** 10.1038/s41597-024-03538-z

**Published:** 2024-06-26

**Authors:** Anna Onisiforou, Panos Zanos, Polymnia Georgiou

**Affiliations:** 1 Department of Psychology, University of Cyprus, Nicosia, Cyprus; 2 Department of Biological Sciences, University of Cyprus, Nicosia, Cyprus; 3 Department of Psychology, University of Wisconsin Milwaukee, Milwaukee, Wisconsin USA

**Keywords:** Psychiatric disorders, Microarrays, Molecular neuroscience

## Abstract

Major depressive disorder (MDD) and substance-use disorders (SUDs) often lead to premature aging, increasing vulnerability to cognitive decline and other forms of dementia. This study utilized advanced systems bioinformatics to identify aging “signatures” in MDD and SUDs and evaluated the potential for known lifespan-extending drugs to target and reverse these signatures. The results suggest that inhibiting the transcriptional activation of *FOS* gene family members holds promise in mitigating premature aging in MDD and SUDs. Conversely, antidepressant drugs activating the PI3K/Akt/mTOR pathway, a common mechanism in rapid-acting antidepressants, may accelerate aging in MDD patients, making them unsuitable for those with comorbid aging-related conditions like dementia and Alzheimer’s disease. Additionally, this innovative approach identifies potential anti-aging interventions for MDD patients, such as Deferoxamine, Resveratrol, Estradiol valerate, and natural compounds like zinc acetate, genistein, and ascorbic acid, regardless of comorbid anxiety disorders. These findings illuminate the premature aging effects of MDD and SUDs and offer insights into treatment strategies for patients with comorbid aging-related conditions, including dementia and Alzheimer’s disease.

## Introduction

Aging is a natural process that occurs in all living organisms and includes the gradual decline in the functions of an organism over time, impacting its physiological systems, functional characteristics, and clinical features^1,2^. It is influenced by genetic, environmental, and behavioral factors, as well as the presence of disease/s^3^. While chronological age is determined by year of birth, biological aging involves a complex interplay of various factors that can result to accelerated aging, leading to the manifestation of aging phenotypes at an earlier stage than typically expected. The operational definitions of aging phenotypes present a challenge, as the existing literature employs diverse age-related biomarkers. Consequently, it becomes imperative to discern the extent to which specific diseases or risk factors contribute to premature aging. Notably, major depressive disorder (MDD) and substance-use disorders (SUDs) are pertinent examples of such conditions warranting investigation.

MDD and SUDs are neuropsychiatric disorders (NPDs) with high prevalence worldwide^4^. Importantly, there is significant comorbidity between the two disorders, with an estimated 30% of individuals diagnosed with MDD also experiencing SUDs. This comorbidity leads to an increased risk of suicide, heightened social and personal dysfunction, and may exacerbate other mental health conditions^5^. Importantly, both MDD and SUDs are associated with premature mortality and early onset of age-related somatic diseases, such as heart disease, diabetes, obesity and cancer^6–11^. There is also strong evidence suggesting the association between NPDs and age-related biological hallmarks, including cellular senescence, mitochondrial dysfunction altered intercellular communication, deregulation of nutrient sensing and epigenetic alternations^6,12^, among others.

Additionally, premature aging of the brain is also observed in individuals suffering from MDD and SUDs. Specifically, individuals suffering from MDD^13^ and SUDs^14,15^ experience early onset dementia and cognitive impairments, prominent features of aging. Pooled magnetic resonance imaging (MRI) data revealed that adults with MDD exhibit advanced brain aging, characterized by increased brain atrophy and decreased brain volume^16^. Moreover, individuals with MDD show accelerated aging of the putamen compared to healthy controls^17^. Similarly, drug abuse accentuates age-related brain changes potentially contributing to abnormalities observed in addiction models. Cocaine-dependent subjects demonstrate an increased rate of brain volume reduction in regions like the prefrontal and temporal cortices compared to healthy controls^18^. Likewise, methamphetamine-dependent individuals exhibit greater-than-normal age-related cortical gray matter loss^19^, and cannabis use is associated with decreased hippocampal volume^20^. Importantly, pathologies commonly observed in the aging brain, such as primary age-related tauopathy^21^ and intra- and extra-cranial artery dysfunction^22^, manifest earlier in individuals with SUDs^6^. Specifically, cocaine^23^, methamphetamine^24^, and heroin^25^ users display intra- and extra-cranial artery dysfunction. Furthermore, cognitive decline in addiction^26^ may be attributed to hyperphosphorylated tau and p62-positive inclusions (neurodegeneration-related proteins) reported with heroin use^27^, or the accumulation of amyloid as reported in cocaine^28^ and methamphetamine^29^ users.

Exploring human aging represents a challenging task, not only because it is a complex phenomenon, but also due to the constraints of studying aging directly in humans which further adds to this difficulty^30,31^. Bioinformatics approach can overcome these limitations and offer an alternative avenue for discovering aging-related mechanisms by integrating and analyzing multidimensional data^30,31^. Additionally, computational methods, such as machine learning and predictive modelling, can identify genes associated with aging^32,33^. While several studies have utilized computational methods to provide valuable insights into the complex processes associated with aging, there is lack of research investigating premature aging in MDD and SUDs.

**Fig. 1:**
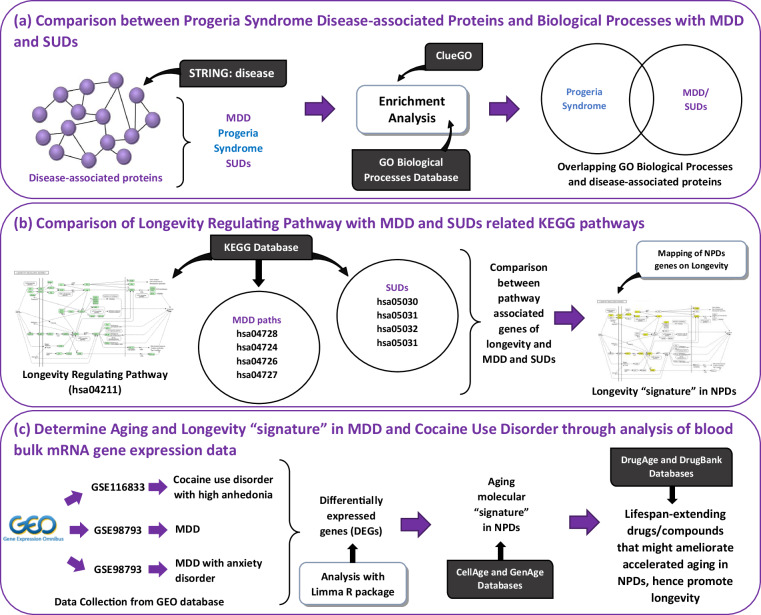
Schematic Illustration of the bioinformatics methodology applied in this paper. (**a**) Comparative analysis of progeria syndrome disease-associated proteins and Gene Ontology biological processes with those of MDD and various SUDs. (**b**) Comparison between genes associated with the KEGG Longevity regulating pathway and the relevant disease KEGG pathways of MDD and SUDs. (**c**) Analysis of blood transcriptomic data from MDD (with and without anxiety disorder) and Cocaine Use Disorder to unveil their aging “signature” and identify known lifespan-extending drugs and compounds that could potentially be used to ameliorate disease-mediated accelerated aging.

Here, we employed an integrated systems bioinformatics approach to elucidate potential mechanisms leading to premature aging in individuals with MDD and SUDs (Fig. 1). To the best of our knowledge, this is the first study to apply a systems bioinformatics methodology in deciphering the aging and longevity “signatures” of NPDs, such as MDD and SUDs. Understanding the major pathophysiological mechanisms through which chronic NPDs contribute to premature aging and decreased longevity is of utmost importance. This insight can facilitate the identification of novel pharmacotherapies for their effective treatment, while also preventing premature aging. Initially, we compared MDD and SUDs (morphine dependence, cocaine dependence, nicotine dependence, and amphetamine abuse) with progeria syndrome, a rare progressive genetic condition characterized by premature aging in children. This comparison allowed us to isolate aging-associated proteins and biological processes whose dysregulation might lead to premature aging in these NPDs (Fig. 1a). Furthermore, we examined the genes associated with the longevity-regulating pathway and compared them with the relevant disease-associated pathways of these NPDs obtained from the Kyoto Encyclopedia of Genes and Genomes (KEGG) database. This part of the analysis aimed to identify longevity-associated genes in these NPDs (Fig. 1b). Additionally, we determined the aging “signatures” of MDD and SUDs by analyzing publicly available whole blood bulk mRNA sequencing data (Fig. 1c). Finally, based on our findings, we compiled a concise list of known lifespan-extending drugs/compounds that interact with the isolated aging “signatures” in individuals with MDD (with or without comorbid anxiety disorder) and SUDs. These identified drugs/compounds have the potential to serve as pharmacotherapies to mitigate accelerated aging in these NPDs (Fig. 1c). Further details on the bioinformatics methodology employed are provided in the Methods section.

## Results

### Comparison of disease-associated proteins and biological processes of Progeria syndrome with Major Depressive Disorder and Substance Use Disorders

To identify disease-associated proteins and pathological mechanisms related to accelerated aging in MDD and various SUDs, we performed a comparative analysis with progeria syndrome, also known as Hutchinson-Gilford progeria syndrome (HGPS). Progeria syndrome is an extremely rare, autosomal dominant disorder that leads to premature and rapid aging in children^34^. Utilizing progeria syndrome as a unique model, characterized by premature aging, allows us to specifically isolate aging-associated genes and biological processes whose dysregulation contributes to premature aging.

**Fig. 2:**
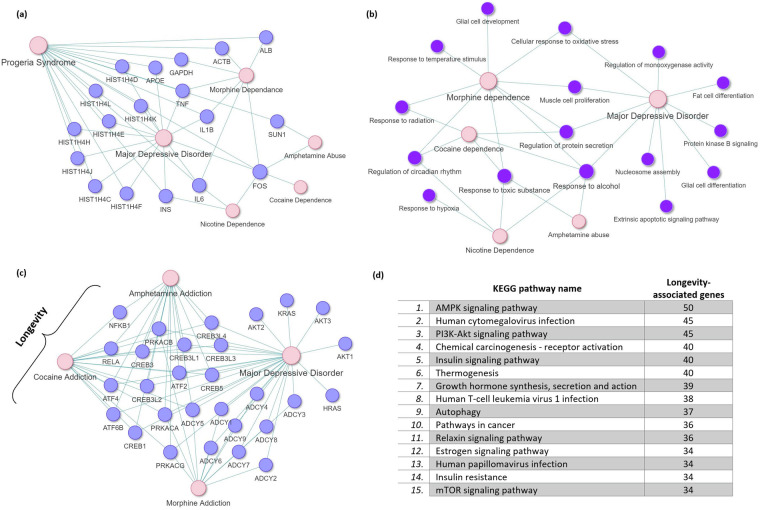
Progeria- and longevity- associated genes in MDD. Common (**a**) disease-associated proteins and (**b**) functional groups of GO biological processes between progeria syndrome and MDD and SUDs. (**c**) Genes associated with the longevity-regulating pathway that are also affected in MDD and SUDs. (**d**) Top 15 KEGG pathways with the highest number of genes associated with Longevity.

Our analysis results revealed that MDD exhibits the highest number of common disease-associated genes with progeria syndrome (Fig. 2a). Intriguingly, the *FOS* gene was found to be common to all five NPDs (MDD, morphine dependence, cocaine dependence, nicotine dependence, and amphetamine dependence) and progeria syndrome (Fig. 2a). *FOS* is a proto-oncogene with crucial roles in cell growth, proliferation, differentiation, transformation, as well as brain development and signal transduction^35,36^. Furthermore, MDD, morphine dependence, and nicotine dependence share pro-inflammatory cytokines interleukin 6 (*IL6*), interleukin 1B (*IL1B*), and tumor necrosis factor (*TNF*) with progeria syndrome (Fig. 2a). Aging is often associated with “inflamm-aging,” characterized by increased levels of blood and tissue pro-inflammatory markers, which pose a risk factor for various age-related diseases, including cardiovascular disease, cognitive decline, and depression^37,38^. Moreover, the presence of inflamm-aging is evidence of the altered intercellular communication which is a hallmark of biological aging and strongly associated with MDD^12^. Therefore, the presence of pro-inflammatory markers in MDD and SUDs serves as a mechanism facilitating accelerated aging in these NPDs. In addition to the presence of inflammatory markers, we identified several markers of epigenetic modifications that are shared with progeria syndrome and MDD (Fig. 2a). Specifically, histone H4 is a protein that in humans is encoded by the *HIST1H4* gene at different loci. Histones are basic nuclear proteins that are responsible for the nucleosome structure of the chromosomal fiber in eukaryotes and are subject to epigenetic modifications like histone acetylation and chromatic remodeling that can alter gene transcription. Importantly, such epigenetic modifications are a hallmark of aging and MDD^12^, and this interaction between progeria syndrome and MDD with regards to histone H4 may indicate that accelerated aging in people with MDD may be due to the increased epigenetic modifications.

Additionally, we conducted a comparison of Gene Ontology (GO) biological processes associated with progeria syndrome and those associated with MDD and four SUDs (morphine dependence, cocaine dependence, nicotine dependence, and amphetamine abuse; see Fig. 1a). The GO biological processes for each condition were determined by performing enrichment analysis on their respective disease-associated proteins (See Methods). Furthermore, functional enrichment analysis was performed to identify the functional groups to which the common processes identified between progeria syndrome and each NPDs belong. The results revealed 37, 17, 8, 7, and 2 common GO biological processes between progeria syndrome and MDD, morphine dependence, cocaine dependence, nicotine dependence, and amphetamine abuse, respectively.

Specifically, functional enrichment analysis of the 37 common GO biological processes shared between progeria syndrome and MDD indicated their association with 10 functional groups (see Fig. 2b and Supplementary File 1: Table 1). Thus, the dysregulation of these identified functional groups in MDD might contribute to accelerated aging. Moreover, the functional enrichment analysis results showed that the common GO biological processes between progeria syndrome and morphine dependence, cocaine dependence, nicotine dependence, and amphetamine abuse belong to 9, 5, 4, and 2 functional groups, respectively (see Fig. 2b and Supplementary File 1: Table 2–5). Although GO analysis could sometimes be agnostic of the direction of the changes in the expression of the genes used as inputs, we have formulated strong priori rationales based on existing literature that informs the interpretation of our findings. Importantly, our analysis revealed processes that are associated with biological aging such as muscle degradation, mitochondrial dysfunction, protein regulation, when comparing progeria syndrome with MDD and SUDs (Fig. 2b). This suggests that these neuropsychiatric disorders may share common underlying mechanisms with progeria syndrome, contributing to accelerated aging processes observed in MDD and SUDs.

### Identification of longevity-associate genes in Major Depressive Disorder and Substance Use Disorders

Longevity, defined as the capability to extend one’s lifespan beyond the average age of death^39^, relies on a combination of genetic and environmental factors that prevent diseases and optimize health^40^. Numerous pathways and processes have been suggested to regulate an organism’s lifespan, thus playing a pivotal role in promoting longevity^41,42^. Consequently, the dysregulation of these pathways and processes by diseases can lead to reduced longevity and accelerate aging.

To identify genes associated with longevity that are also implicated in MDD and SUDs, we conducted a comparison between the genes associated with the KEGG Longevity regulating pathway (hsa04211) and the relevant disease KEGG pathways of these NPDs. Initially, using the KEGG database, we extracted the genes associated with each of the relevant KEGG pathways, resulting in a collection of 89 genes associated with the Longevity regulating pathway. Additionally, we collected 49, 69, 91, and 40 genes associated with the Cocaine addiction (hsa05030), Amphetamine addiction (hsa05031), Morphine addiction (hsa05032), and Nicotine addiction (hsa05033) pathways, respectively. As for MDD, no directly related pathway was available in the KEGG database. Therefore, we selected four main pathways (Dopaminergic synapse (hsa04728), Glutamatergic synapse (hsa04724), Serotonergic synapse (hsa04726), and GABAergic synapse (hsa04727)) that have been implicated in MDD, serving as reference points^43–46^. Overall, after eliminating duplicate entries, we assembled a total of 288 genes associated with the four pathways representing MDD.

Comparison with the Longevity regulating pathway revealed 16, 16, and 12 common genes with SUDs (Cocaine addiction, Amphetamine addiction, and Morphine addiction, respectively) (Fig. 2c). Additionally, comparing the 89 genes associated with the Longevity regulating pathway with the 288 genes associated with the four pathways that act as reference pathways for MDD revealed 28 common genes (Fig. 2c). However, no common genes were found between the Longevity regulating pathway and the Nicotine addiction pathway. Furthermore, mapping the longevity-associated genes found in MDD and the SUDs onto the Longevity regulating pathway indicates that in all four NPDs, some of these genes participate in the PI3K-Akt signaling pathway (**see** Supplementary File 1: Figs. 1–3).

We also explored other pathways contained in the KEGG database that involve genes associated with the Longevity regulating pathway, as several of these pathways are implicated in the pathophysiology of MDD and SUDs. After parsing the KEGG database, we identified 196 Homo sapiens pathways that include at least one gene associated with the Longevity regulating pathway. Among these, the top 15 KEGG pathways with the highest number of longevity-associated genes are indicated in Fig. 2d. Remarkably, the AMPK signaling pathway stood out with the highest number of 50 genes associated with the Longevity regulating pathway. AMP-activated protein kinase (AMPK) plays a crucial role in the regulation of energy metabolism, nutrient sensing and cellular homeostasis, as well as in conferring resistance to stress and promoting autophagy^47^. Studies have demonstrated that increased AMPK activation extends lifespan and mitigates or halts the aging process^48–50^. Conversely, decreased responsiveness of AMPK activation during aging leads to various physiological changes that accelerate aging, such as reduced autophagy, heightened inflammation and fat deposition, hyperglycemia, deregulation of nutrient sensing and enhanced metabolic syndrome^12,49^. Interestingly, inactivation of the AMPK signaling pathway has also been associated with depressive-like behaviors, which can be ameliorated through pharmacological interventions promoting AMPK signaling activation^51,52^. Moreover, there is considerable evidence connecting the development of depression with aging through the deregulation of nutrient sensing via the AMPK signaling pathway^12^.

The top 15 pathways also include the Estrogen and mTOR signaling pathways, both of which are implicated in MDD and SUDs^53–56^. Additionally, it encompasses three viral infection pathways: Human cytomegalovirus infection, Human T-cell leukemia virus 1 infection, and Human papillomavirus infection. Neurotropic viruses, like human cytomegalovirus (HCMV), which infect the brain, can lead to accelerated brain aging by interfering with microglia function, thus promoting low-grade neuroinflammation and contributing to “inflamm-aging”^57^. In the context of NPDs, drugs and depression can weaken the immune system, making individuals more susceptible to viral infections or leading to the reactivation of latent viruses within the human host. Consequently, the body’s compromised ability to mount an effective immune response against new viral threats or to suppress reactivated viruses may result in viral-mediated accelerated aging in these NPDs^58,59^.

### Aging molecular “signatures” in Major Depressive Disorder and Cocaine Use Disorder (CUD) based on blood mRNA sequencing data

To further characterize the aging molecular “signatures” in MDD (with and without anxiety disorder) and Cocaine Use Disorder (CUD) that contribute to accelerated aging, we analyzed publicly available peripheral blood samples obtained from the Gene Expression Omnibus database (GEO). Transcriptomic data for other SUDs is not publicly available in the GEO database. As a result, we are presenting only results for CUD in this part of the analysis. The aim was to identify aging-associated differentially expressed genes (DEGs) in these NPDs. This was accomplished by comparing their DEGs with experimentally validated aging-associated gene expression patterns (see Methods).

### Aging expression patterns in Neuropsychiatric Disorders

#### Major Depressive Disorder

**Fig. 3:**
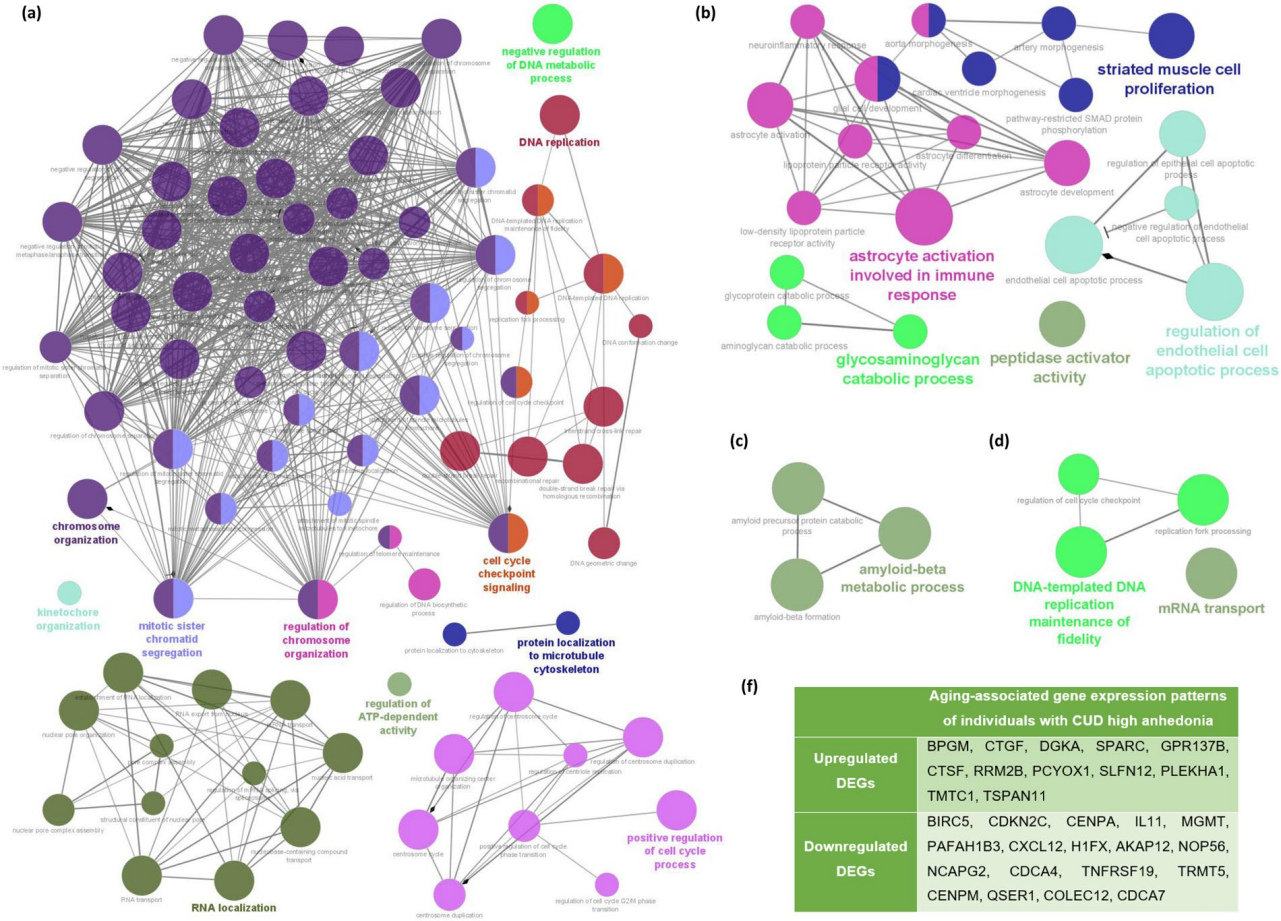
GO biological processes associated with aging in NPDs. Biological processes associated with the (**a**) downregulated and (**b**) upregulated aging-associated DEG patterns in MDD, as well as the (**c**) upregulated and (**d**) downregulated aging-associated DEG patterns in MDD with comorbid anxiety disorder. (**f**) Up-and down-regulated aging-associated DEG patterns of CUD with high anhedonia.

Comparison of the DEGs determined from blood samples of MDD patients (GSE98793) with the experimentally validated aging-associated gene expression patterns collected from CellAge and GenAge databases^60^, revealed 81 DEGs that are also found to be upregulated in aging and 92 DEGs that are also downregulated in aging. Enrichment analysis of the downregulated aging-associated DEGs in MDD revealed that they are associated with 85 statistically significantly enriched GO Biological Processes terms that belong to eleven functional groups (Fig. 3a). Additionally, the upregulated aging-associated DEGs in MDD are associated with 21 GO biological processes that belong to five functional groups (Fig. 3b). The identified up- and down-regulated biological processes could represent possible pathological mechanisms that accelerate aging in MDD.

#### Major Depressive Disorder with comorbid Anxiety Disorder

Comparison of the DEGs determined from the analysis of blood samples from MDD patients with comorbid anxiety disorder (GSE98793) with the experimentally validated aging-associated gene expression patterns revealed 41 upregulated and 41 downregulated DEGs in MDD that are also associated with aging. Enrichment analysis of the upregulated aging-associated DEGs revealed that they are associated with 3 statistically significant GO biological processes that belong to the functional group of amyloid-beta metabolic process (Fig. 3c) which is in line with published work^12,61^ that helped formed our prior hypothesis. In addition, the downregulated aging-associated DEGs were found to be associated with 4 statistically significantly enriched GO biological processes that belong to two functional groups (Fig. 3d).

#### Cocaine Use Disorder with high anhedonia

Anhedonia, defined as the inability to feel pleasure or joy, is a central feature of MDD and addictive disorders, such as CUD^62,63^. By analyzing the gene expression patterns of blood samples (GSE116833) from individuals with CUD with high anhedonia (n = 24) compared to those with low anhedonia (n = 24), we determined the DEG patterns specific to CUD with high anhedonia. Comparison of the DEGs from individuals with CUD with high anhedonia with the experimentally validated aging-associated gene expression patterns revealed 12 upregulated and 18 downregulated genes associated with aging (Fig. 3f). However, enrichment analysis of the up- and down-regulated aging-associated DEGs did not reveal any statistically significantly enriched GO biological processes.

### Longevity expression patterns in Neuropsychiatric Disorders

#### Major depressive disorder

To determine the longevity gene expression patterns in MDD, we compared the MDD DEGs (GSE98793) with the 89 genes associated with the KEGG Longevity regulating pathway. This comparison revealed 14 upregulated and 10 downregulated DEGs in MDD associated with the KEGG Longevity regulating pathway (Table 1). Subsequently, mapping the longevity-associated DEGs of MDD on the Longevity regulating pathway (map04211) indicated that the identified DEGs participate in the Insulin signaling pathway, PI3K-Akt signaling pathway, mTOR signaling pathway, FOXO signaling pathway, and AMPK signaling pathway (**see** Supplementary File 1: Fig. 4).

**Table 1:** Differentially expressed gene patterns associated with the KEGG Longevity regulating pathway in neuropsychiatric disorders.

	Upregulated longevity-associated DEGs	Downregulated longevity- associated DEGs
MDD	INS, INSR, IGF1R, IRS2, PIK3R2, ADCY1, ADCY5, PRKACA, CREB5, FOXO3, RHEB, ULK1, RPS6KB2, CAMKK2	KRAS, PIK3CA, PIK3R1, AKT3, PRKACB, CREB3L2, APPL1, PRKAB2, PRKAG2, CAMK4
MDD with comorbid anxiety disorder	INSR, IGF1R, IRS1, IRS2, ADCY7, FOXO3, EIF4E, PRKAA1, PRKAA2, SESN3	PIK3R1, MTOR, EIF4E2, PRKAG2
CUD with high anhedonia	EHMT1, SOD2	CREB3L1, CREB5, AKT1S1, ULK1

#### Major Depressive Disorder with comorbid Anxiety Disorder

Comparison of the DEGs patterns of MDD with comorbid anxiety disorder (GSE98793) revealed 10 upregulated and 4 downregulated DEGs associated with the KEGG Longevity regulating pathway (Table 1). Mapping of the longevity-associated DEGs of MDD with comorbid anxiety disorder on the Longevity regulating pathway map indicates that they participate in the same pathways as MDD without comorbid anxiety disorder (**see** Supplementary File 1**:** Fig. 5).

#### Cocaine Use Disorder with high anhedonia

Comparison of the DEGs patterns of individuals with CUD with high anhedonia (GSE116833) revealed 2 upregulated and 4 downregulated DEGs associated with the KEGG Longevity regulating pathway (Table 1). Mapping on the Longevity regulating pathway map indicates that the longevity-associated DEGs of CUD with high anhedonia participate in the PI3K-Akt signaling pathway, mTOR signaling pathway, and FOXO signaling pathway (**see** Supplementary File 1**:** Fig. 6).

### Lifespan-extending drugs and compounds that might reduce accelerated aging in Neuropsychiatric Disorders

By using the isolated aging molecular “signatures” of the NPDs, we have identified a list of potential lifespan-extending drugs/compounds, that have been experimentally shown to extend lifespan based on model organisms^64^. These identified drugs/compounds hold promise as potential therapeutic interventions to reduce accelerated aging in these NPDs. By counteracting the effects of the DEGs determined through our analysis to promote accelerated aging in these NPDs, these drugs/compounds have the potential to promote longevity. For MDD, we have identified 10 lifespan-extending drugs/compounds (Table 2) that target the aging-associated DEGs.

**Table 2:** Lifespan-extending drugs/compounds that might reduce accelerated aging in major depressive disorder, major depressive disorder with comorbid anxiety disorder and cocaine use disorder with high anhedonia.

Disease	Drug Name	Disease DEGs Drug target	Aging Signature of DEGs in Disease
MDD	Ascorbic acid	PLOD1	upregulated in aging
	Folic acid	FOLR3	upregulated in aging
	Zinc acetate	FN1	upregulated in aging
	Ascorbic acid	PLOD2	upregulated in aging
	Deferoxamine	APP	upregulated in aging
	Resveratrol	APP	upregulated in aging
	Zinc acetate	APP	upregulated in aging
	Chloroquine	HMGB1	downregulated in aging
	Genistein	GPER1	upregulated in aging
	Estradiol valerate	GPER1	upregulated in aging
MDD with comorbid anxiety disorder	Ascorbic acid	PLOD2	upregulated in aging
	Deferoxamine	APP	upregulated in aging
	Resveratrol	APP	upregulated in aging
	Zinc acetate	APP	upregulated in aging
	Genistein	GPER1	upregulated in aging
	Estradiol valerate	GPER1	upregulated in aging
CUD with high anhedonia	Cysteine	MGMT	downregulated in aging
	Zinc acetate	MGMT	downregulated in aging
	Vitamin E	DGKA	upregulated in aging
	Reserpine	BIRC5	downregulated in aging
	Berberine	BIRC5	downregulated in aging

In addition, we identified 6 lifespan-extending drugs/compounds (Table 2) that have the potential to prevent accelerated aging in MDD with comorbid anxiety disorder by modulating the aging-associated DEGs. Finally, we have identified 5 lifespan-extending drugs/compounds (Table 2) that have the potential to prevent accelerated aging in individuals with CUD with high anhedonia compared to those with low anhedonia.

## Discussion

This groundbreaking study utilizes an integrated systems bioinformatics approach to characterize the aging and longevity molecular “signatures” of MDD and various SUDs. The primary aim of this study was to isolate possible mechanisms that might facilitate premature aging and decrease longevity in individuals with these NPDs. We have identified specific disease-associated genes and biological processes shared between progeria syndrome and these NPDs. These findings shed light on possible pathways leading to accelerated aging. Furthermore, we have identified specific genes associated with longevity that are also affected in MDD and SUDs. Additionally, we have identified the top KEGG pathways containing the highest number of longevity-associated genes. The transcriptomic molecular “signatures” of aging in MDD, MDD with comorbid anxiety disorder, and CUD with high anhedonia have also been characterized through the analysis of publicly available mRNA peripheral blood samples. Finally, this study has also highlighted specific known lifespan-extending drugs/compounds that interact with the isolated transcriptomic molecular “signatures” of aging in these NPDs. These identified drugs/compounds may have the potential to reduce or ameliorate accelerated aging in individuals with these NPDs, serving as promising anti-aging prophylactic treatments.

More specifically, we have found compelling evidence that MDD exhibits the highest number of common disease-associated genes and GO biological processes with progeria syndrome. Notably, the *FOS* gene has been identified as a common disease-associated gene shared between progeria syndrome and all five NPDs (MDD, morphine dependence, cocaine dependence, amphetamine abuse, and nicotine dependence). Moreover, the *FOS* gene participates in several of the common biological processes found between progeria syndrome and these NPDs. The *FOS* gene encodes a family of transcription factors responsible for regulating gene expression in response to various stimuli. One particular member, *ΔFosB*, has garnered significant interest due to its role in drug addiction and depression^65^. Following repeated exposure to drugs of abuse or stress, ΔFosB accumulates in the brain and remains stable for extended periods, altering the expression of genes involved in synaptic plasticity, reward, and mood regulation. Consequently, this contributes to the long-term behavioral changes observed in addiction and depression, such as increased sensitivity to drugs, enhanced drug-seeking behavior, and maintaining the addiction cycle^66–70^. Notably, transcriptional activation of the *FOS* gene has been identified as a conserved aging signature across tissues, contributing to the phenomenon of “inflamm-aging”^71^. Consistent with this observation, we noted common proinflammatory cytokines, such as *IL6, IL1B*, and *TNF*, shared between progeria syndrome with MDD and Morphine Dependence, as well as *IL6* with Nicotine dependence. Increased inflammation is a hallmark of aging and is associated with a higher risk of developing cardiovascular disease, cognitive decline, and depression^37,38^. It is important to highlight that chronic exposure to stimuli, such as substance misuse and stress, not only induces *ΔFosB* but also leads to increased neuroinflammation, which contributes to the development of substance use and mood disorders^70,72^. In line with this, we observed that the cellular response to oxidative stress is a common GO for biological processes between morphine dependence, MDD and progeria syndrome, which in addition to mitochondrial dysfunction indicates impairment in inflammatory processes. Therefore, targeting the transcriptional activation of *FOS* gene family members might hold promise as a therapeutic strategy to alleviate symptoms of NPDs and, importantly, to prevent the premature aging often observed in these individuals.

Moreover, we identified significant genetic overlap between MDD and progeria syndrome, specifically involving genes associated with epigenetic modifications. One notable example is histone H4. Interestingly, overexpression of histone H3/H4 in yeast extends the lifespan, suggesting that an increased pool of free histones promotes survival during aging^73^ and the reduction in histone acetylation plays a critical role in aging-related chromatin remodeling, resulting in the condensation and suppression of genes linked to neuroprotection and synaptic plasticity among others^74^. In line with this, histone deacetylase inhibitors have antidepressant effects and stress-induced depressive phenotype is associated with decreased histone acetylation^75,76^. Altogether these findings suggest that histone deacetylation may be a promising approach for the treatment of MDD in premature aging.

Our data further reveal that the MDD (Dopaminergic synapse, Glutamatergic synapse, Serotonergic synapse, GABAergic synapse) and SUD (Morphine addiction, Cocaine addiction, Amphetamine addiction) related KEGG pathways include genes associated with the KEGG Longevity regulating pathway. Notably, several of these longevity-associated genes are found within the PI3K-Akt signaling pathway, as highlighted by their mapping on the KEGG Longevity regulating pathway. Interestingly, the PI3K-Akt signaling pathway emerges as the third KEGG pathway involving the highest number of longevity-associated genes. The PI3K-Akt signaling pathway is a pivotal regulator of diverse cellular processes, including survival, growth, metabolism, and plasticity^77^. Moreover, this pathway plays a critical role in the pathophysiology and treatment of various mental disorders, such as depression and addiction. It achieves this through several mechanisms, including the modulation of synaptic transmission, neuroplasticity, and neuroinflammation^78–81^.

Moreover, the PI3K-Akt signaling pathway can also modulate the activity of other cellular pathways related to depression and addiction, such as the mammalian target of rapamycin (mTOR) signaling pathway^53,54^. Interestingly, the mTOR signaling pathway was also among the top 15 KEGG pathways containing the highest number of longevity-associated genes. mTOR is a kinase that regulates protein synthesis and cell growth^82^. It has been implicated in the regulation of synaptic plasticity, neurogenesis, and the mechanism of action of some antidepressant drugs^83^. Evidence from animal models and human postmortem brain tissues indicates that depression is associated with a reduction in the PI3K/Akt/mTOR signaling in brain regions like the prefrontal cortex (PFC) and amygdala^84,85^. Some antidepressant drugs exert their effects by activating the PI3K/Akt/mTOR pathway. For example, ketamine, a glutamate NMDA receptor antagonist, can rapidly activate this pathway in multiple brain regions, leading to synaptogenesis and antidepressant effects^86^. Other antidepressant drugs that activate the PI3K/Akt/mTOR pathway include valproic acid^87^, AZD6765^88^, escitalopram, and paroxetine^89^.

Interestingly, the activity of *Akt* was found to be upregulated in the aging brain, while downregulation of *Akt* in neurons was shown to reduce Aβ toxicity and improve cell survival^90^, indicating its potential role in modulating aging-related processes and neurodegenerative conditions like Alzheimer’s disease (AD). Pharmacological inhibition of the *Akt* signaling pathway has also been demonstrated to extend lifespan in model organisms like *Dorsophila* and mice^91,92^. Furthermore, decreased activity of the PI3K/Akt/mTOR signaling has been associated with promoting longevity^93^. Activation of the PI3K/Akt signaling pathway can also promote the production of pro-inflammatory cytokines and inhibit anti-inflammatory cytokines, thus contributing to inflamm-aging^94^. Given that repeated or prolonged use of some antidepressant drugs have been associated with an increased risk of dementia and AD^95,96^, these aging-associated pathologies could be mediated through increased activation of the PI3K/Akt/mTOR signaling, as its activation promotes aging pathophysiology, whereas its inhibition promotes longevity. Therefore, accelerated aging in depression might not only arise from disease pathophysiology but also from certain pharmacotherapies used for its treatment. Considering the contrasting functions of PI3K/Akt/mTOR in depression and aging, it will be interesting to conduct further experimental investigations to elucidate their exact roles and functions in the development of NPDs and accelerated aging.

Most importantly, our analysis highlighted a short-list of candidate antiaging interventions from a list of 207 lifespan-extending drugs/compounds that could possibly reduce accelerated aging in MDD, MDD with comorbid anxiety disorder, and CUD with high anhedonia. Specifically, our analysis revealed 6 common drugs/compounds that could potentially reverse the aging-associated molecular “signatures” of both MDD without and with comorbid anxiety disorder. These drugs include Deferoxamine, Resveratrol, and zinc acetate, as well as the natural compounds Estradiol valerate, genistein, and ascorbic acid. Deferoxamine, Resveratrol, and zinc acetate target the amyloid precursor protein (*APP)* gene, which is upregulated in aging and was also found to be upregulated in both MDD with and without comorbid anxiety disorder through our analysis. *APP* is the primary protein involved in the pathogenesis of Alzheimer’s disease (AD)^97^. Interestingly, it has been shown that MDD increases the susceptibility risk for the later development of AD^98^ However, the precise mechanism by which MDD increases AD comorbidity risk remains unclear. AD is the most common form of dementia in the elderly, and its early stages exhibit characteristics of accelerated aging^99^.

Based on our results from the isolated aging-associated molecular “signature” of MDD, *APP* participates in biological processes involved in the upregulation of astrocyte activation and immune responses. On the other hand, MDD with comorbid anxiety disorder, it is involved in the upregulation of biological processes related to amyloid-beta metabolic processes. Thus, *APP* exerts different pathological effects in MDD compared to MDD with anxiety disorder. Consequently, although the identified drugs/compounds Deferoxamine, Resveratrol, and zinc acetate target the *APP* gene in both conditions, they are likely to exert their anti-aging effects through different mechanisms.

Additionally, Estradiol valerate was also identified as a potential lifespan-extending drug for both MDD with and without anxiety disorder, by targeting the G protein-coupled estrogen receptor 1 (*GPER1)* gene. The estrogen signaling pathway was also found to be among the top 15 KEGG pathways containing the highest number of genes associated with the KEGG Longevity regulating pathway. It is well known that the estrogen signaling pathway undergoes several changes during aging due to the reduction in the production of gonadal hormones in both men and women^100^. Moreover, several lines of evidence suggest that the estradiol signaling pathway influences depression and SUDs by modulating reward, motivation, learning, memory, stress, and emotion^101^. Administration of estradiol in women, as well as in both male and female animals, has been shown to result in antidepressant effects, suggesting the potential therapeutic implications of targeting this pathway for the treatment of depression^102–105^. These findings collectively indicate that targeting the estradiol system might not only be beneficial for ameliorating the pathophysiology associated with these NPDs, but also for reducing accelerated aging, possibly by targeting the *GPER1* gene. Apart from Estradiol valerate, our analysis also indicates that the natural compound Genistein, which is a phytoestrogen found in various vegetables such as soybeans, and structurally resembles estrogen, also targets the *GPER1* gene. Therefore, Genistein could represent an alternative natural therapeutic and antiaging option. However, it is worth noting that the effect of estrogens on longevity and the incidence of age-related diseases is still not well understood and these results need to be further investigated to determine their exact role in NPDs and premature aging.

However, our analysis is not without limitations. Firstly, utilizing cross-sectional datasets means that data is collected at a single point in time from different individuals. This approach may not capture the dynamic nature of aging processes, as it does not track changes in individuals over time. Consequently, the findings may lack temporal resolution and may not fully represent the longitudinal progression of aging-related changes. Additionally, cross-sectional datasets may introduce biases due to cohort effects, selection bias, or confounding variables, which can affect the generalizability and validity of the results. Another limitation is the lack of external validation sets of the computationally the identified lifespan-extending drugs/compounds with the possible potential to reduce accelerated aging in NPDs. The lifespan-extending drugs/compounds utilized in our analysis have long been recognized for their potential anti-aging effects, as evidenced by numerous studies in various animal models. However, their efficacy in humans is still under investigation, particularly concerning their anti-aging effects under different disease phenotypes. The lifespan-extending drugs/compounds highlighted through our analysis are based on the aging signature of the NPDs under investigation. Consequently, these compounds possess a unique mechanism of action that targets pathways implicated in accelerated aging specifically associated with these NPDs. We anticipate that our proposed candidate lifespan-extending drugs/compounds will emerge as promising candidates for future research and clinical application to validate their effectiveness in preventing accelerated aging in individuals with NPDs.

Despite these limitations, our research yields invaluable insights regarding the aging molecular signature of MDD and SUDs. Characterizing the aging and longevity “signatures” of NPDs, will not only aid in identifying pathophysiological mechanisms that accelerate aging in these disorders but also enable the identification of potential anti-aging interventions, as highlighted by our analysis. This approach can open novel research directions for the development of prophylactic treatments to reduce the risk of developing comorbid dementia or AD, which might arise from the disease itself or certain pharmacological interventions used for the treatment of these NPDs. Additionally, our findings underscore the importance of considering biological age and the presence of comorbid aging-associated diseases as factors when selecting antidepressant treatments in older individuals. Some of these treatments may exacerbate aging, potentially accelerating the pathophysiology of diseases such as dementia and AD. Therefore, it is crucial to carefully evaluate the potential effects of antidepressant medications on aging processes and age-related conditions to ensure safer and more effective treatment options for older individuals with mental disorders.

Future research should prioritize longitudinal studies to provide a more comprehensive understanding of the dynamic changes in aging-related processes over time and their association with MDD and SUDs. Furthermore, pre-clinical and clinicals studies are needed to evaluate the efficacy and safety of potential interventions, such as inhibiting the transcriptional activation of *FOS* gene family members or administering lifespan-extending drugs like Deferoxamine and Resveratrol, in reducing accelerated aging in MDD and SUD patients. These trials should also assess the impact of these interventions on cognitive function, quality of life, and other relevant outcomes. Additionally, future research should explore the underlying mechanisms through which antidepressant drugs activating the PI3K/Akt/mTOR pathway accelerate aging in MDD patients. Understanding these mechanisms could lead to the development of safer and more effective antidepressant treatments that do not exacerbate premature aging. Moreover, efforts should be made to translate these findings into clinical practice by integrating personalized medicine approaches. Tailoring interventions based on individual aging signatures and disease phenotypes could optimize treatment outcomes and improve the overall well-being of patients with MDD, SUDs, and comorbid aging-related conditions. Overall, these findings provide valuable insights into the premature aging effects of MDD and SUDs and offer promising directions for future research.

## Methods

### Datasets

The mRNA seq datasets GSE116833 and GSE98793 used for this study can be found in the Gene Expression Omnibus database (https://www.ncbi.nlm.nih.gov/geo/).

Additional data used in this study were:

DrugAge database (https://genomics.senescence.info/drugs/);

CellAge database (https://genomics.senescence.info/cells/);

GenAge database (https://genomics.senescence.info/genes/index.html);

KEGG database (https://www.genome.jp/kegg/pathway.html);

DrugBank (https://go.drugbank.com/);

### Comparison between Progeria syndrome disease-associated proteins and biological processes with MDD and SUDs

#### Disease-associated proteins

Our aim was to compare the disease-associated proteins and biological processes of progeria syndrome with those of MDD and various SUDs with the aim to identify the presence of aging-associated genes and processes in these NPDs. We used a methodology developed in our previous works^106–108^. First, we utilized the *String: disease app*^109^ in Cytoscape to extract the top 200 disease-associated proteins with the highest disease score. This involved configuring the maximum number of protein parameters to 200. The *STRING: disease app* sources its data from the DISEASES database (https://diseases.jensenlab.org/Search). This database compiles gene-disease associations from a variety of evidence types, which are subsequently consolidated and assigned a confidence score. A high confidence gene-to-disease association is represented by 5 stars, while a low confidence association is indicated by 1 star, signifying the likelihood of it being a true positive. We collected data for Progeria syndrome (DOID: 3911) and the following five NPDs: MDD (DOID: 1470), morphine dependence (DOID: 2560), cocaine dependence (DOID: 9975), nicotine dependence (DOID: 0050742), and amphetamine abuse (DOID: 670). Subsequently, we compared the disease-associated proteins of progeria syndrome with each of the five NPDs. However, it’s important to note that for amphetamine abuse, only 182 disease-associated proteins were considered instead of 200, as the database did not provide additional associated proteins for this condition.

#### Enrichment analysis

We also conducted enrichment analysis on the 200 disease-associated proteins of each condition to identify their related biological processes. The enrichment analysis was performed using the *ClueGO* app^110^ in Cytoscape, where only significantly enriched terms with an adjusted p-value ≤ 0.01 (corrected with Bonferroni step-down) were retained in the GO biological processes category. Subsequently, we compared the GO biological processes of progeria syndrome with each of the five NPDs, aiming to isolate common biological processes shared with progeria syndrome. The objective was to identify pathological mechanisms that might lead to premature aging in these NPDs. Additionally, functional enrichment analysis was performed on the identified common GO biological processes (Progeria ∩ NPDs) using the preselected function of the *ClueGO* app. This analysis enabled us to identify the functional groups to which these biological processes belong. The preselected function in ClueGO helps visualize and group GO terms as a functionally grouped network, where the interrelations (edges) between terms and functional groups are determined based on shared genes between terms.

### Comparison of KEGG Longevity regulating pathway with NPDs related KEGG pathways

#### MDD and SUDs related KEGG pathways

To investigate whether MDD and SUDs involve genes associated with longevity, we used the KEGG database to compare the Longevity regulating pathway (hsa04211) with the relevant disease-associated KEGG pathways of these NPDs. Using the KEGGREST^111^ package in R, we parsed the KEGG database^112^ and extracted the names of all 352 KEGG pathways found in the database. From these 352 pathways, we identified 4 pathways that relate to drug addiction. However, the KEGG database did not contain any pathway specific to MDD. Therefore, in the case of MDD, the four KEGG pathways Dopaminergic synapse (hsa04728), Glutamatergic synapse (hsa04724), Serotonergic synapse (hsa04726), and GABAergic synapse (hsa04727) were selected to act as its reference points. We chose these four pathways to represent the MDD “pathway” because there is extensive evidence that dysregulation of these four pathways is associated with its development^43–46^. Using the KEGGREST package, we collected from the KEGG database the genes associated with each of the following pathways: Longevity regulating pathway (hsa04211), Cocaine addiction (hsa05030), Amphetamine addiction (hsa05031), Morphine addiction (hsa05032), Nicotine addiction (hsa05033), Dopaminergic synapse (hsa04728), Glutamatergic synapse (hsa04724), Serotonergic synapse (hsa04726), and GABAergic synapse (hsa04727). We then compared the genes associated with the KEGG Longevity regulating pathway with the relevant NPDs KEGG pathways to identify common associated genes of these NPDs with the longevity pathway. For MDD, we combined the associated genes found from the four KEGG pathways (Dopaminergic synapse, Glutamatergic synapse, Serotonergic synapse, GABAergic synapse) that were selected to act as disease “reference points” and compared them with the Longevity regulating pathway-associated genes. Furthermore, we mapped the identified longevity genes of each NPDs on the Longevity regulating pathway map (map04211) to highlight the longevity-related pathways that might be affected.

#### Longevity associated genes in other KEGG pathways

Additionally, using the KEGGREST package in R^111^, we identified which of the 352 KEGG pathways found in the KEGG database contain at least one gene associated with the  Longevity regulating pathway, in order to identify the KEGG pathways that contain the highest number of genes associated with longevity.

### Determining the aging and longevity molecular “signatures” of MDD and SUDs through analysis of whole blood bulk mRNA sequencing data

#### Collection of blood mRNA sequencing data

To determine the gene expression patterns, including upregulation or downregulation, of genes associated with aging and longevity in MDD and SUDs, we first analyzed publicly available bulk mRNA sequencing data. The GEO database^113^, which contains transcriptomic data, was used to retrieve blood gene expression studies of MDD and SUDs. The following search terms were used: “major depressive disorder,” “Nicotine,” “Nicotine addiction,” “Amphetamine,” “Amphetamine addiction,” “Cocaine addiction,” “Cocaine,” “Morphine,” and “Morphine addiction.” Only microarray studies with (i) a control group, (ii) 15 experimental group samples or more, and (iii) the biosample was blood were selected for analysis. Three studies were identified to meet our inclusion criteria: (i) Dataset with ID GSE116833 that contains data for Cocaine addiction and includes 48 experimental cases, of which 24 are individuals with CUD with high anhedonia and 24 CUD with low anhedonia, (ii) dataset GSE98793 that contains data for MDD and includes 64 experimental cases and 64 healthy controls, and (iii) dataset GSE98793 that contains data for MDD with comorbid anxiety disorder and includes 64 experimental cases and 64 healthy controls.

#### Data Pre-Processing and identification of DEGs

All three datasets were pre-processed and analyzed in the R environment for statistical analysis and visualization^114^. Each dataset was normalized and log2 transformed. The Limma R package^115^, which allows the identification of DEGs from bulk mRNA sequencing data, was then used to analyze each dataset. The probe-set IDs were then matched to each corresponding gene symbol based on each platform’s annotations file. In the case of multiple probe-set IDs for the same gene symbol, the average value was calculated and maintained for the gene symbol. Only genes with a *p-*value below 0.05 were considered statistically significant and were used for further analysis.

### Comparison of aging- and longevity- associated genes with the DEG patterns of NPDs

Experimentally validated aging-associated gene expression patterns were obtained from CellAge and GenAge databases^60^. Overall, we collected 1259 genes associated with aging, of which 255 were found to be upregulated and 734 downregulated in aging. We then performed a comparison analysis between the aging-associated gene expression patterns with the DEGs of MDD, MDD with anxiety, and CUD with high anhedonia to determine the aging molecular “signatures” of these NPDs. Positive genes were defined as genes found in both aging and the NPD, as well as having the same differential expression pattern (upregulated or downregulated). Whereas negative genes were defined as genes found only in one condition or both but had the opposite differential expression pattern, i.e., upregulated in aging and downregulated in the NPD. We also performed enrichment analysis to determine the biological processes to which the positive genes belong. Enrichment analysis was performed separately on the positively correlated upregulated and downregulated genes, using the same enrichment method described above.

In addition, to determine the longevity gene expression patterns of these NPDs, we compared their DEGs with the 89 genes associated with the KEGG Longevity regulating pathway. We then mapped the identified longevity DEGs of each NPD on the Longevity regulating pathway map (map04211) to highlight the longevity-related pathways that might be affected.

### Identification of lifespan-extending drugs and compounds that might reduce accelerated aging in NPDs

The DrugAge database^64^ was used to collect known lifespan-extending drugs and compounds that were experimentally validated to extend lifespan in model organisms. Overall, we collected 1096 lifespan-extending drugs/compounds from the DrugAge database. Then, by using the DrugBank database^116^, we collected their drug/compound-gene interactions, where we identified 1739 drug/compound-to-gene interactions between 207 lifespan-extending drugs/compounds and 1020 gene targets.

Next, we compared the 1020 gene targets of the 207 lifespan-extending drugs/compounds with the aging molecular “signatures” found for each NPD (MDD, MDD with comorbid anxiety disorder, and CUD with high anhedonia) through the analysis of bulk mRNA data. The aim was to isolate lifespan-extending drugs/compounds that interact with the aging-associated DEGs and, thus, could possibly ameliorate accelerated aging or promote longevity in these NPDs. A shortlist of candidate lifespan-extending drugs/compounds was isolated for each NPD.

### Supplementary information

Supplementary information

## Data Availability

The analysis results associated with this study are available on Figshare^117^ (10.6084/m9.figshare.c.6901717.v1).

## Code availability

All of the programs we use in this study for analysis are free and open-access software. No custom code was used during this study for the curation and/or validation of the dataset.

Information on the version of the open-source software and its official website:

1. Cytoscape (v3.8.2) https://cytoscape.org/

2. stringAPP (v2.0.1) https://apps.cytoscape.org/apps/stringapp

3. ClueGO (v2.5.10) https://apps.cytoscape.org/apps/cluego

4. KEGGREST (v1.42.0) https://bioconductor.org/packages/release/bioc/html/KEGGREST.html

5. Limma (v3.18) https://bioconductor.org/packages/release/bioc/html/limma.html

## References

[1] Tartiere, A. G., Freije, J. M. P. & López-Otín, C. The hallmarks of aging as a conceptual framework for health and longevity research. *Front. Aging* **5** (2024). 10.3389/fragi.2024.1334261 PMC10824251 38292053

[2] López-Otín C, Blasco MA, Partridge L, Serrano M, Kroemer G (2023). Hallmarks of aging: An expanding universe. Cell.

[3] Pac A (2019). Influence of Sociodemographic, Behavioral and Other Health-Related Factors on Healthy Ageing Based on Three Operative Definitions. J. Nutr. Heal. Aging.

[4] Castelpietra, G. *et al*. The burden of mental disorders, substance use disorders and self-harm among young people in Europe, 1990–2019: Findings from the Global Burden of Disease Study 2019. *Lancet Reg. Heal. - Eur*. **16** (2022). 10.1016/j.lanepe.2022.100341 PMC8980870 35392452

[5] Davis L, Uezato A, Newell JM, Frazier E (2008). Major depression and comorbid substance use disorders. Current Opinion in Psychiatry.

[6] Bachi K, Sierra S, Volkow ND, Goldstein RZ, Alia-Klein N (2017). Is biological aging accelerated in drug addiction?. Current Opinion in Behavioral Sciences.

[7] Ojo, O., Wang, X. H., Ojo, O. O. & Ibe, J. The effects of substance abuse on blood glucose parameters in patients with diabetes: A systematic review and meta-analysis. *International Journal of Environmental Research and Public Health* vol. 15 (2018). 10.3390/ijerph15122691 PMC6313386 30501025

[8] Sansone RA, Sansone LA (2013). Obesity and substance misuse: Is there a relationship?. Innov. Clin. Neurosci..

[9] Nicholson A, Kuper H, Hemingway H (2006). Depression as an aetiologic and prognostic factor in coronary heart disease: A meta-analysis of 6362 events among 146 538 participants in 54 observational studies. Eur. Heart J..

[10] Mezuk B, Eaton WW, Albrecht S, Golden SH (2008). Depression and type 2 diabetes over the lifespan: A meta-analysis. Diabetes Care.

[11] Luppino FS (2010). Overweight, obesity, and depression: A systematic review and meta-analysis of longitudinal studies. Archives of General Psychiatry.

[12] Lorenzo, E. C., Kuchel, G. A., Kuo, C. L., Moffitt, T. E. & Diniz, B. S. Major depression and the biological hallmarks of aging. *Ageing Research Reviews* vol. 83 (2023). 10.1016/j.arr.2022.101805 PMC9772222 36410621

[13] Rosness TA, Barca ML, Engedal K (2010). Occurrence of depression and its correlates in early onset dementia patients. Int. J. Geriatr. Psychiatry.

[14] Hulse, G. K., Lautenschlager, N. T., Tait, R. J. & Almeida, O. P. Dementia associated with alcohol and other drug use. *International Psychogeriatrics* vol. 17 (2005). 10.1017/s1041610205001985 16240487

[15] Gould TJ (2010). Addiction and cognition. Addiction science & clinical practice.

[16] Han LKM (2021). Brain aging in major depressive disorder: results from the ENIGMA major depressive disorder working group. Mol. Psychiatry.

[17] Sacchet MD, Camacho MC, Livermore EE, Thomas EAC, Gotlib IH (2017). Accelerated aging of the putamen in patients with major depressive disorder. J. Psychiatry Neurosci..

[18] Ersche KD, Jones PS, Williams GB, Robbins TW, Bullmore ET (2013). Cocaine dependence: A fast-track for brain ageing. Molecular Psychiatry.

[19] Nakama H (2011). Methamphetamine users show greater than normal age-related cortical gray matter loss. Addiction.

[20] Battistella G (2014). Long-term effects of cannabis on brain structure. Neuropsychopharmacology.

[21] Crary JF (2014). Primary age-related tauopathy (PART): a common pathology associated with human aging. Acta Neuropathol..

[22] Gutierrez J (2016). Brain arterial aging and its relationship to Alzheimer dementia. Neurology.

[23] Massardo T (2015). Changes in regional cerebral blood flow are associated with endothelial dysfunction markers in cocaine-dependent patients under recent abstinence. J. Addict. Med..

[24] Ho EL, Josephson SA, Lee HS, Smith WS (2009). Cerebrovascular complications of methamphetamine abuse. Neurocrit. Care.

[25] Benoilid A, Collongues N, de Seze J, Blanc F (2013). Heroin inhalation-induced unilateral complete hippocampal stroke. Neurocase.

[26] Sanvicente-Vieira B, Kommers-Molina J, de Nardi T, Francke I, Grassi-Oliveira R (2016). Crack-cocaine dependence and aging: Effects on working memory. Rev. Bras. Psiquiatr..

[27] Kovacs GG (2015). Heroin abuse exaggerates age-related deposition of hyperphosphorylated tau and p62-positive inclusions. Neurobiol. Aging.

[28] Shvartsbeyn M (2010). Cocaine-induced intracerebral hemorrhage in a patient with cerebral amyloid angiopathy. J. Forensic Sci..

[29] Wallace TL, Vorhees CV, Zemlan FP, Gudelsky GA (2003). Methamphetamine enhances the cleavage of the cytoskeletal protein tau in the rat brain. Neuroscience.

[30] de Magalhães JP, Toussaint O (2004). How bioinformatics can help reverse engineer human aging. Ageing Res. Rev..

[31] Dato S, Crocco P, Rambaldi Migliore N, Lescai F (2021). Omics in a Digital World: The Role of Bioinformatics in Providing New Insights Into Human Aging. Front. Genet..

[32] Kulaga, A. Y. *et al*. Machine Learning Analysis of Longevity-Associated Gene Expression Landscapes in Mammals. *Int. J. Mol. Sci*. **22** (2021). 10.3390/ijms22031073 PMC7865694 33499037

[33] Fabris F, Palmer D, Salama KM, de Magalhães JP, Freitas AA (2020). Using deep learning to associate human genes with age-related diseases. Bioinformatics.

[34] Ahmed MS, Ikram S, Bibi N, Mir A (2018). Hutchinson–Gilford Progeria Syndrome: A Premature Aging Disease. Molecular Neurobiology.

[35] Verma IM, Mitchell RL, Sassone-Corsi P (1986). Proto-oncogene fos: an inducible gene. Princess Takamatsu symposia.

[36] Cruz-Mendoza, F., Jauregui-Huerta, F., Luquin, S., Aguilar-Delgadillo, A. & García-Estrada, J. Immediate Early Gene c-fos in the Brain: Focus on Glial Cells. *Brain Sci*. **12** (2022). 10.3390/brainsci12060687 PMC9221432 35741573

[37] Michaud M (2013). Proinflammatory cytokines, aging, and age-related diseases. Journal of the American Medical Directors Association.

[38] Ferrucci L, Fabbri E (2018). Inflammageing: chronic inflammation in ageing, cardiovascular disease, and frailty. Nature Reviews Cardiology.

[39] De Benedictis, G. & Franceschi, C. The unusual genetics of human longevity. *Science of aging knowledge environment: SAGE KE* vol. 2006 (2006). 10.1126/sageke.2006.10.pe20 16807484

[40] Brooks-Wilson AR (2013). Genetics of healthy aging and longevity. Human Genetics.

[41] Acosta-Rodríguez V (2022). Circadian alignment of early onset caloric restriction promotes longevity in male C57BL/6J mice. Science (80-.)..

[42] Mutlu AS, Duffy J, Wang MC (2021). Lipid metabolism and lipid signals in aging and longevity. Developmental Cell.

[43] Saez E (2022). Genetic variables of the glutamatergic system associated with treatment-resistant depression: A review of the literature. World J. Psychiatry.

[44] Duman RS, Sanacora G, Krystal JH (2019). Altered Connectivity in Depression: GABA and Glutamate Neurotransmitter Deficits and Reversal by Novel Treatments. Neuron.

[45] Pourhamzeh M (2022). The Roles of Serotonin in Neuropsychiatric Disorders. Cellular and Molecular Neurobiology.

[46] Kapur S, John Mann J (1992). Role of the dopaminergic system in depression. Biological Psychiatry.

[47] Garcia D, Shaw RJ (2017). AMPK: Mechanisms of Cellular Energy Sensing and Restoration of Metabolic Balance. Molecular Cell.

[48] Stancu AL (2015). AMPK activation can delay aging. Discoveries.

[49] Salminen A, Kaarniranta K (2012). AMP-activated protein kinase (AMPK) controls the aging process via an integrated signaling network. Ageing Research Reviews.

[50] Ge Y, Zhou M, Chen C, Wu X, Wang X (2022). Role of AMPK mediated pathways in autophagy and aging. Biochimie.

[51] Yuan, S. Y. *et al*. AMPK mediates glucocorticoids stress-induced downregulation of the glucocorticoid receptor in cultured rat prefrontal cortical astrocytes. *PLoS One* **11** (2016). 10.1371/journal.pone.0159513 PMC4981361 27513844

[52] Ren, Y. *et al*. Juglanin ameliorates depression-like behavior in chronic unpredictable mild stress-induced mice by improving AMPK signaling. *J. Funct. Foods* **98** (2022).

[53] Ignácio, Z. M. *et al*. New perspectives on the involvement of mTOR in depression as well as in the action of antidepressant drugs. *British Journal of Clinical Pharmacology* 1280–1290, 10.1111/bcp.12845 (2016). 10.1111/bcp.12845 PMC5061805 26613210

[54] Ucha M, Roura-Martínez D, Ambrosio E, Higuera-Matas A (2020). The role of the mTOR pathway in models of drug-induced reward and the behavioural constituents of addiction. Journal of Psychopharmacology.

[55] Kokane SS, Perrotti LI (2020). Sex Differences and the Role of Estradiol in Mesolimbic Reward Circuits and Vulnerability to Cocaine and Opiate Addiction. Front. Behav. Neurosci..

[56] Georgiou, P., Zanos, P., Jenne, C. E. & Gould, T. D. Sex-Specific Involvement of Estrogen Receptors in Behavioral Responses to Stress and Psychomotor Activation. *Front. Psychiatry* **10** (2019). 10.3389/fpsyt.2019.00081 PMC6399411 30863326

[57] Filgueira, L., Larionov, A. & Lannes, N. The influence of virus infection on microglia and accelerated brain aging. *Cells* vol. 10 (2021). 10.3390/cells10071836 PMC8303900 34360004

[58] Zhang HG (2022). Depression compromises antiviral innate immunity via the AVP-AHI1-Tyk2 axis. Cell Res..

[59] Friedman H, Newton C, Klein TW (2003). Microbial infections, immunomodulation, and drugs of abuse. Clinical Microbiology Reviews.

[60] Tacutu R (2018). Human Ageing Genomic Resources: New and updated databases. Nucleic Acids Res..

[61] dos Santos HM (2024). Dementia and depression: Biological connections with amyloid β protein. Basic Clin. Pharmacol. Toxicol..

[62] Destoop, M., Morrens, M., Coppens, V. & Dom, G. Addiction, anhedonia, and comorbid mood disorder. A narrative review. *Frontiers in Psychiatry* vol. 10 (2019). 10.3389/fpsyt.2019.00311 PMC6538808 31178763

[63] Heininga, V. E. *et al*. The dynamical signature of anhedonia in major depressive disorder: Positive emotion dynamics, reactivity, and recovery. *BMC Psychiatry* **19** (2019). 10.1186/s12888-018-1983-5 PMC6368777 30736751

[64] Barardo D (2017). The DrugAge database of aging-related drugs. Aging Cell.

[65] Volkow ND, Michaelides M, Baler R (2019). The neuroscience of drug reward and addiction. Physiol. Rev..

[66] Nestler EJ (2008). Transcriptional mechanisms of addiction: Role of ΔFosB. Philos. Trans. R. Soc. B Biol. Sci..

[67] Nestler EJ (2015). δfosB: A transcriptional regulator of stress and antidepressant responses. Eur. J. Pharmacol..

[68] Vialou V (2010). ΔfosB in brain reward circuits mediates resilience to stress and antidepressant responses. Nat. Neurosci..

[69] Nestler EJ, Barrot M, Self DW (2001). ΔFosB: A sustained molecular switch for addiction. Proc. Natl. Acad. Sci. USA.

[70] Lobo MK (2013). ΔFosB induction in striatal medium spiny neuron subtypes in response to chronic pharmacological, emotional, and optogenetic stimuli. J. Neurosci..

[71] Karakaslar, E. O. *et al*. Transcriptional activation of Jun and Fos members of the AP-1 complex is a conserved signature of immune aging that contributes to inflammaging. *Aging Cell* **22** (2023). 10.1111/acel.13792 PMC10086525 36840360

[72] Flores-López, M. *et al*. Inflammatory factors and depression in substance use disorder. in *The Neuroscience of Depression: Genetics, Cell Biology, Neurology, Behavior, and Diet* 149–160, 10.1016/B978-0-12-817935-2.00025-8 (2021).

[73] Wang K (2022). Epigenetic regulation of aging: implications for interventions of aging and diseases. Signal Transduct. Target. Ther..

[74] Vitorakis, N. & Piperi, C. Insights into the Role of Histone Methylation in Brain Aging and Potential Therapeutic Interventions. *International Journal of Molecular Sciences* vol. 24 (2023). 10.3390/ijms242417339 PMC10744334 38139167

[75] Yuan M (2023). Epigenetic regulation in major depression and other stress-related disorders: molecular mechanisms, clinical relevance and therapeutic potential. Signal Transduct. Target. Ther..

[76] Park, H.-S., Kim, J., Ahn, S. H. & Ryu, H.-Y. Epigenetic Targeting of Histone Deacetylases in Diagnostics and Treatment of Depression. *Int. J. Mol. Sci*. **22** (2021). 10.3390/ijms22105398 PMC8160658 34065586

[77] Fruman DA (2017). The PI3K Pathway in Human Disease. Cell.

[78] Kitagishi, Y., Kobayashi, M., Kikuta, K. & Matsuda, S. Roles of PI3K/AKT/GSK3/mTOR pathway in cell signaling of mental illnesses. *Depression Research and Treatment* vol. 2012 (2012). 10.1155/2012/752563 PMC3535741 23320155

[79] Liu HQ (2019). Critical roles of the PI3K-Akt-mTOR signaling pathway in apoptosis and autophagy of astrocytes induced by methamphetamine. Open Chem..

[80] Beaulieu JM (2012). A role for Akt and glycogen synthase kinase-3 as integrators of dopamine and serotonin neurotransmission in mental health. Journal of Psychiatry and Neuroscience.

[81] Matsuda S (2019). Roles of PI3K/AKT/GSK3 Pathway Involved in Psychiatric Illnesses. Diseases.

[82] Saxton RA, Sabatini DM (2017). Erratum: mTOR Signaling in Growth, Metabolism, and Disease (Cell (2017) 168(6) (960–976) (S0092867417301824) (10.1016/j.cell.2017.02.004)). Cell.

[83] Dwyer JM, Duman RS (2013). Activation of mammalian target of rapamycin and synaptogenesis: Role in the actions of rapid-acting antidepressants. Biol. Psychiatry.

[84] Chandran A (2013). Reduced phosphorylation of the mTOR signaling pathway components in the amygdala of rats exposed to chronic stress. Prog. Neuro-Psychopharmacology Biol. Psychiatry.

[85] Jernigan CS (2011). The mTOR signaling pathway in the prefrontal cortex is compromised in major depressive disorder. Prog. Neuro-Psychopharmacology Biol. Psychiatry.

[86] Duman RS, Li N, Liu RJ, Duric V, Aghajanian G (2012). Signaling pathways underlying the rapid antidepressant actions of ketamine. in Neuropharmacology.

[87] Lima IVdA (2017). Antidepressant-like effect of valproic acid—Possible involvement of PI3K/Akt/mTOR pathway. Behav. Brain Res..

[88] Neis, V. B. *et al*. The involvement of PI3K/Akt/mTOR/GSK3β signaling pathways in the antidepressant-like effect of AZD6765. *Pharmacol. Biochem. Behav*. **198** (2020). 10.1016/j.pbb.2020.173020 32861641

[89] Seo, M. K. *et al*. Effects of escitalopram and paroxetine on mTORC1 signaling in the rat hippocampus under chronic restraint stress. *BMC Neurosci*. **18** (2017). 10.1186/s12868-017-0357-0 PMC5405541 28446154

[90] Chen, Y. R. *et al*. Aging-induced Akt activation involves in aging-related pathologies and Aβ-induced toxicity. *Aging Cell* **18** (2019). 10.1111/acel.12989 PMC6612704 31183966

[91] Cheng, X. *et al*. Inhibitor GSK690693 extends Drosophila lifespan via reduce AKT signaling pathway. *Mech. Ageing Dev*. **202** (2022). 10.1016/j.mad.2022.111633 35065134

[92] Selvarani R, Mohammed S, Richardson A (2021). Effect of rapamycin on aging and age-related diseases—past and future. GeroScience.

[93] Lamming DW (2014). Diminished mTOR signaling: a common mode of action for endocrine longevity factors. SpringerPlus.

[94] Vergadi E, Ieronymaki E, Lyroni K, Vaporidi K, Tsatsanis C (2017). Akt Signaling Pathway in Macrophage Activation and M1/M2 Polarization. J. Immunol..

[95] Yan J, Huang Y, Lu Y, Chen J, Jiang H (2014). Repeated administration of ketamine can induce hippocampal neurodegeneration and long-term cognitive impairment via the ROS/HIF-1α pathway in developing rats. Cell. Physiol. Biochem..

[96] Wang, Y. C. *et al*. Increased Risk of Dementia in Patients with Antidepressants: A Meta-Analysis of Observational Studies. *Behavioural Neurology* vol. 2018 (2018). 10.1155/2018/5315098 PMC6079596 30123386

[97] O’Brien RJ, Wong PC (2011). Amyloid precursor protein processing and alzheimer’s disease. Annu. Rev. Neurosci..

[98] Green RC (2003). Depression as a risk factor for Alzheimer disease: The MIRAGE Study. Arch. Neurol..

[99] Leparulo, A. *et al*. Accelerated Aging Characterizes the Early Stage of Alzheimer’s Disease. *Cells* **11** (2022). 10.3390/cells11020238 PMC8774248 35053352

[100] Horstman AM, Dillon EL, Urban RJ, Sheffield-Moore M (2012). The role of androgens and estrogens on healthy aging and longevity. Journals of Gerontology - Series A Biological Sciences and Medical Sciences.

[101] Hwang WJ, Lee TY, Kim NS, Kwon JS (2021). The role of estrogen receptors and their signaling across psychiatric disorders. International Journal of Molecular Sciences.

[102] Hernández-Hernández OT, Martínez-Mota L, Herrera-Pérez JJ, Jiménez-Rubio G (2018). Role of Estradiol in the Expression of Genes Involved in Serotonin Neurotransmission: Implications for Female Depression. Curr. Neuropharmacol..

[103] Herson M, Kulkarni J (2022). Hormonal Agents for the Treatment of Depression Associated with the Menopause. Drugs and Aging.

[104] Carrier N (2015). The Anxiolytic and Antidepressant-like Effects of Testosterone and Estrogen in Gonadectomized Male Rats. Biol. Psychiatry.

[105] Benmansour, S., Arroyo, L. D. & Frazer, A. Comparison of the antidepressant-like effects of estradiol and that of selective serotonin reuptake inhibitors in middle-aged ovariectomized rats. *Front. Aging Neurosci*. **8** (2016). 10.3389/fnagi.2016.00311 PMC5174113 28066235

[106] Onisiforou A, Spyrou GM (2022). Systems Bioinformatics Reveals Possible Relationship between COVID-19 and the Development of Neurological Diseases and Neuropsychiatric Disorders. Viruses.

[107] Onisiforou, A. & Spyrou, G. M. Immunomodulatory effects of microbiota-derived metabolites at the crossroad of neurodegenerative diseases and viral infection: network-based bioinformatics insights. *Front. Immunol*. **13** (2022). 10.3389/fimmu.2022.843128 PMC9344014 35928817

[108] Onisiforou A, Spyrou GM (2021). Identification of viral-mediated pathogenic mechanisms in neurodegenerative diseases using network-based approaches. Brief. Bioinform..

[109] Szklarczyk D (2021). The STRING database in 2021: Customizable protein-protein networks, and functional characterization of user-uploaded gene/measurement sets. Nucleic Acids Res..

[110] Bindea G (2009). ClueGO: A Cytoscape plug-in to decipher functionally grouped gene ontology and pathway annotation networks. Bioinformatics.

[111] Tenenbaum D & Maintainer B. KEGGREST: Client-side REST access to the Kyoto Encyclopedia of Genes and Genomes (KEGG). R package version 1.38.0. (2022).

[112] Kanehisa M, Goto S (2000). KEGG: Kyoto Encyclopedia of Genes and Genomes. Nucleic Acids Res..

[113] Edgar R, Domrachev M, Lash AE (2002). Gene Expression Omnibus: NCBI gene expression and hybridization array data repository. Nucleic Acids Res..

[114] Ihaka R, Gentleman RR (1996). A Language for Data Analysis and Graphics. J. Comput. Graph. Stat..

[115] Ritchie ME (2015). limma powers differential expression analyses for RNA-sequencing and microarray studies. Nucleic Acids Res..

[116] Wishart DS (2018). DrugBank 5.0: A major update to the DrugBank database for 2018. Nucleic Acids Res..

[117] Onisiforou A, Zanos P, Georgiou P (2024). Figshare.

