# Supplementary material for: Molecular signatures of premature aging in Major Depression and Substance Use Disorders
Source: Sci Data. 2024 Jun 26;11:698. doi: 10.1038/s41597-024-03538-z (PMC11208564; doi:10.1038/s41597-024-03538-z)
Supplement: Supplementary file 1 — Supplementary information [file 41597_2024_3538_MOESM1_ESM.docx]

**Supplementary Information**

**Aging and Longevity Molecular Signatures of Major Depression and Substance Use Disorders**

**Anna Onisiforou, Panos Zanos, Polymnia Georgiou**

Table of Contents

[**Supplementary Table 1:** Functional groups of common GO Biological processes of Progeria Syndrome with MDD. 2](#_Toc168854854)

[**Supplementary Table 2:** Functional groups of common GO Biological processes of Progeria Syndrome with Nicotine Dependence. 2](#_Toc168854855)

[**Supplementary Table 3:** Functional groups of common GO Biological processes of Progeria Syndrome with Morphine dependence. 2](#_Toc168854856)

[**Supplementary Table 4:** Functional groups of common GO Biological processes of Progeria Syndrome with Cocaine dependence. 3](#_Toc168854857)

[**Supplementary Table 5:** Functional groups of common GO Biological processes of Progeria Syndrome with Amphetamine abuse. 3](#_Toc168854858)

[**Supplementary Figure 1**: Mapping of the longevity-common genes found with the MDD related KEGG pathways 4](#_Toc168854859)

[**Supplementary Figure 2**: Mapping of the longevity-common genes found with the Morphine addiction pathway 5](#_Toc168854860)

[**Supplementary Figure 3**: Mapping of the longevity-common genes found with the Cocaine and Amphetamine addiction pathways 6](#_Toc168854861)

[**Supplementary Figure 4**: Mapping of the longevity-associated DEGs of MDD 7](#_Toc168854862)

[**Supplementary Figure 5:** Mapping of the longevity-associated DEGs of MDD with comorbid anxiety disorder 8](#_Toc168854863)

[**Supplementary Figure 6:** Mapping of the longevity-associated DEGs of CUD with high anhedonia 9](#_Toc168854864)

# **Supplementary Table 1:** Functional groups of common GO Biological processes of Progeria Syndrome with MDD.

| Functional groups of GO Biological Processes | % Terms per group |
| --- | --- |
| Nucleosome assembly | 36.36 |
| Regulation of monooxygenase activity | 18.18 |
| Glial cell differentiation | 15.15 |
| Muscle cell proliferation | 9.09 |
| Cellular response to oxidative stress | 6.06 |
| Response to alcohol | 3.03 |
| Extrinsic apoptotic signaling pathway | 3.03 |
| Regulation of protein secretion | 3.03 |
| Fat cell differentiation | 3.03 |
| Protein kinase B signaling | 3.03 |

# **Supplementary Table 2:** Functional groups of common GO Biological processes of Progeria Syndrome with Nicotine Dependence.

| Functional groups of GO Biological Processes | % Terms per group |
| --- | --- |
| Response to hypoxia | 40.0 |
| Response to toxic substance | 20.0 |
| Regulation of circadian rhythm | 20.0 |
| Response to alcohol | 20.0 |

# **Supplementary Table 3:** Functional groups of common GO Biological processes of Progeria Syndrome with Morphine dependence.

| Functional groups of GO Biological Processes | % Terms per group |
| --- | --- |
| Muscle cell proliferation | 28.57 |
| Response to radiation | 14.29 |
| Response to alcohol | 14.29 |
| Regulation of circadian rhythm | 7.14 |
| Cellular response to oxidative stress | 7.14 |
| Glial cell development | 7.14 |
| Response to toxic substance | 7.14 |
| Regulation of protein secretion | 7.14 |
| Response to temperature stimulus | 7.14 |
|  |  |

# **Supplementary Table 4:** Functional groups of common GO Biological processes of Progeria Syndrome with Cocaine dependence.

| Functional groups of GO Biological Processes | % Terms per group |
| --- | --- |
| Response to radiation | 33.33 |
| Response to alcohol | 16.67 |
| Regulation of protein secretion | 16.67 |
| Regulation of circadian rhythm | 16.67 |
| Response to toxic substance | 16.67 |
|  |  |

# **Supplementary Table 5:** Functional groups of common GO Biological processes of Progeria Syndrome with Amphetamine abuse.

| Functional groups of GO Biological Processes | % Terms per group |
| --- | --- |
| Response to alcohol | 50.0 |
| Response to toxic substance | 50.0 |

# **Supplementary Figure 1**: Mapping of the longevity-common genes found with the MDD related KEGG pathways


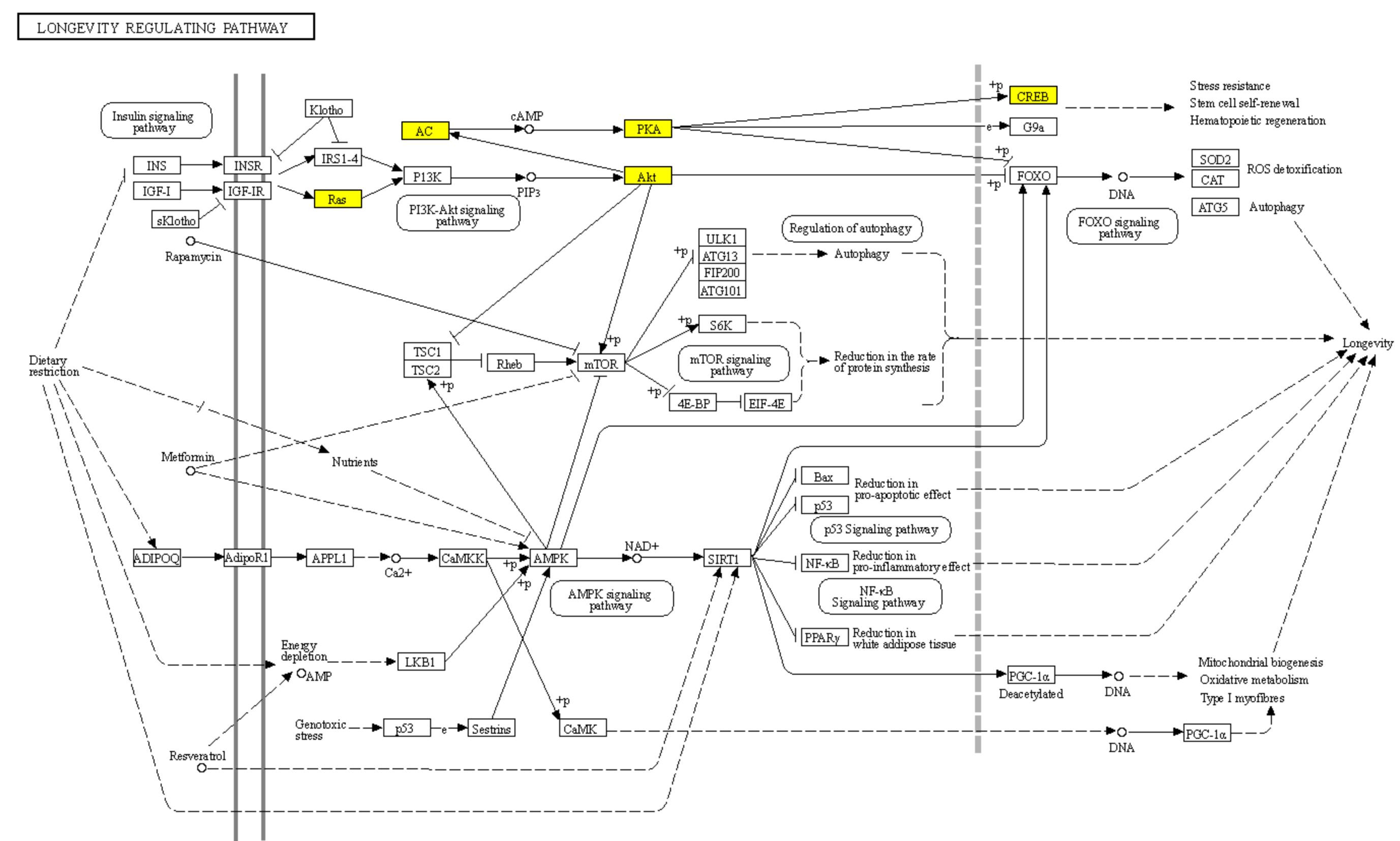


**Supplementary Figure 1:** Mapping of the longevity-common genes found with the MDD related KEGG pathways (Dopaminergic synapse, Glutamatergic synapse, Serotonergic synapse, GABAergic synapse), highlighted in yellow color, on the Longevity regulating pathway.

# **Supplementary Figure 2**: Mapping of the longevity-common genes found with the Morphine addiction pathway


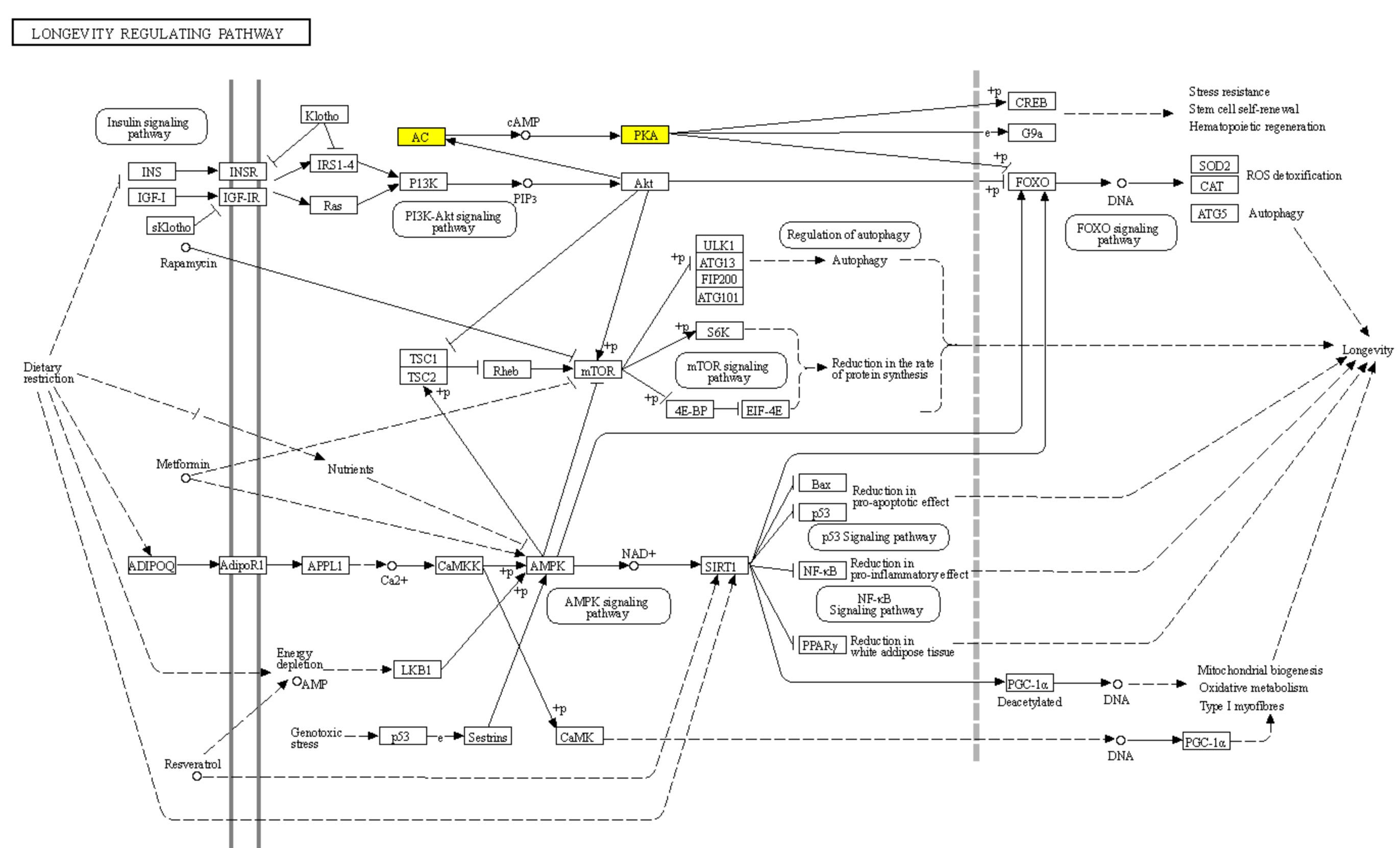
­­­

**Supplementary Figure 2:** Mapping of the longevity-common genes found with the Morphine addiction pathway, highlighted in yellow color, on the Longevity regulating pathway.

# **Supplementary Figure 3**: Mapping of the longevity-common genes found with the Cocaine and Amphetamine addiction pathways


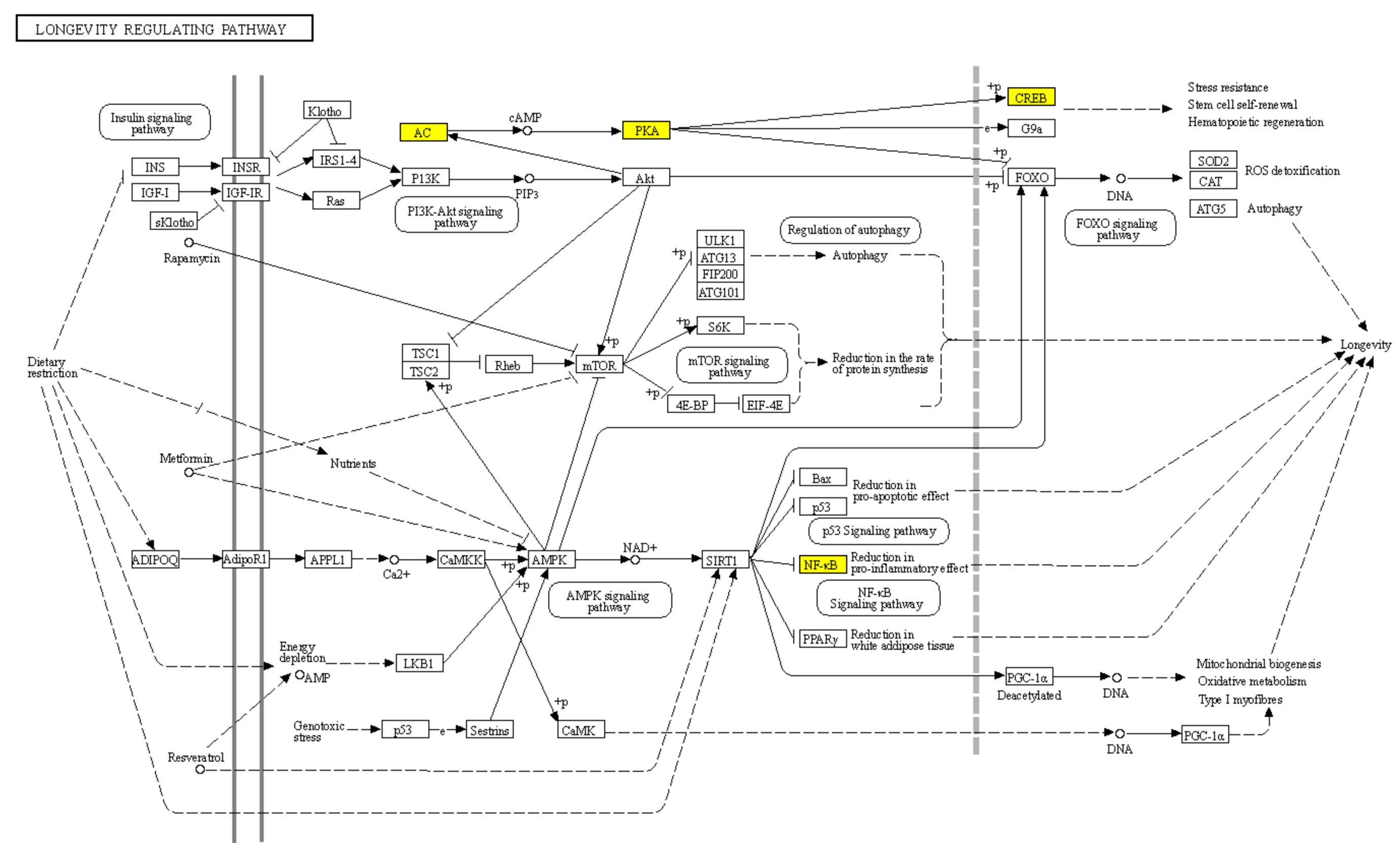


**Supplementary Figure 3:** Mapping of the longevity-common genes found with the Cocaine and Amphetamine addiction pathways, highlighted in yellow color, on the Longevity regulating pathway.

#
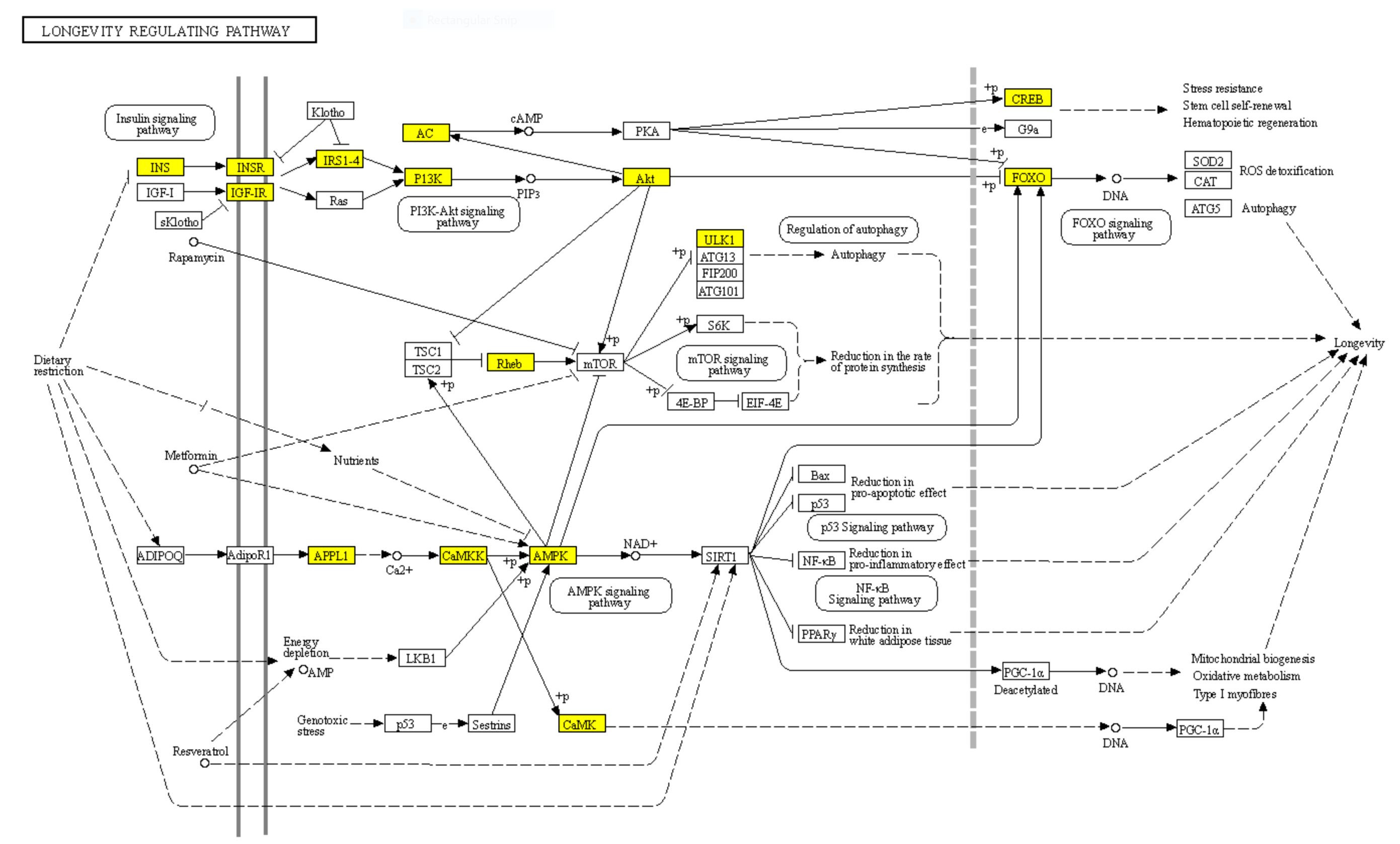
**Supplementary Figure 4**: Mapping of the longevity-associated DEGs of MDD

**Supplementary Figure 4:** Mapping of the longevity-associated DEGs of MDD, highlighted in yellow color, on the KEGG Longevity regulating pathway.

# **Supplementary Figure 5:** Mapping of the longevity-associated DEGs of MDD with comorbid anxiety disorder


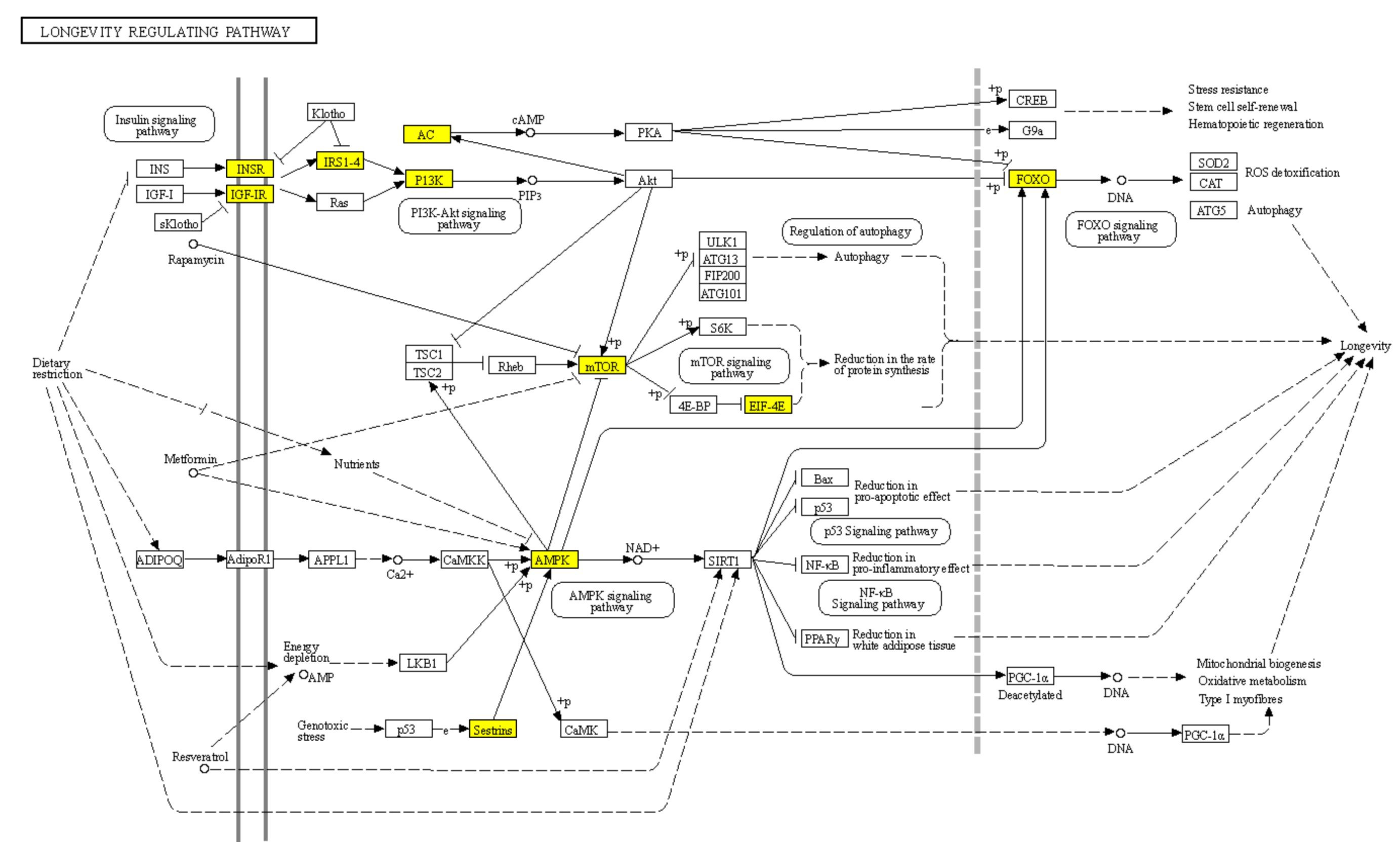


**Supplementary Figure 5:** Mapping of the longevity-associated DEGs of MDD with comorbid anxiety disorder, highlighted in yellow color, on the KEGG Longevity regulating pathway.

# **Supplementary Figure 6:** Mapping of the longevity-associated DEGs of CUD with high anhedonia


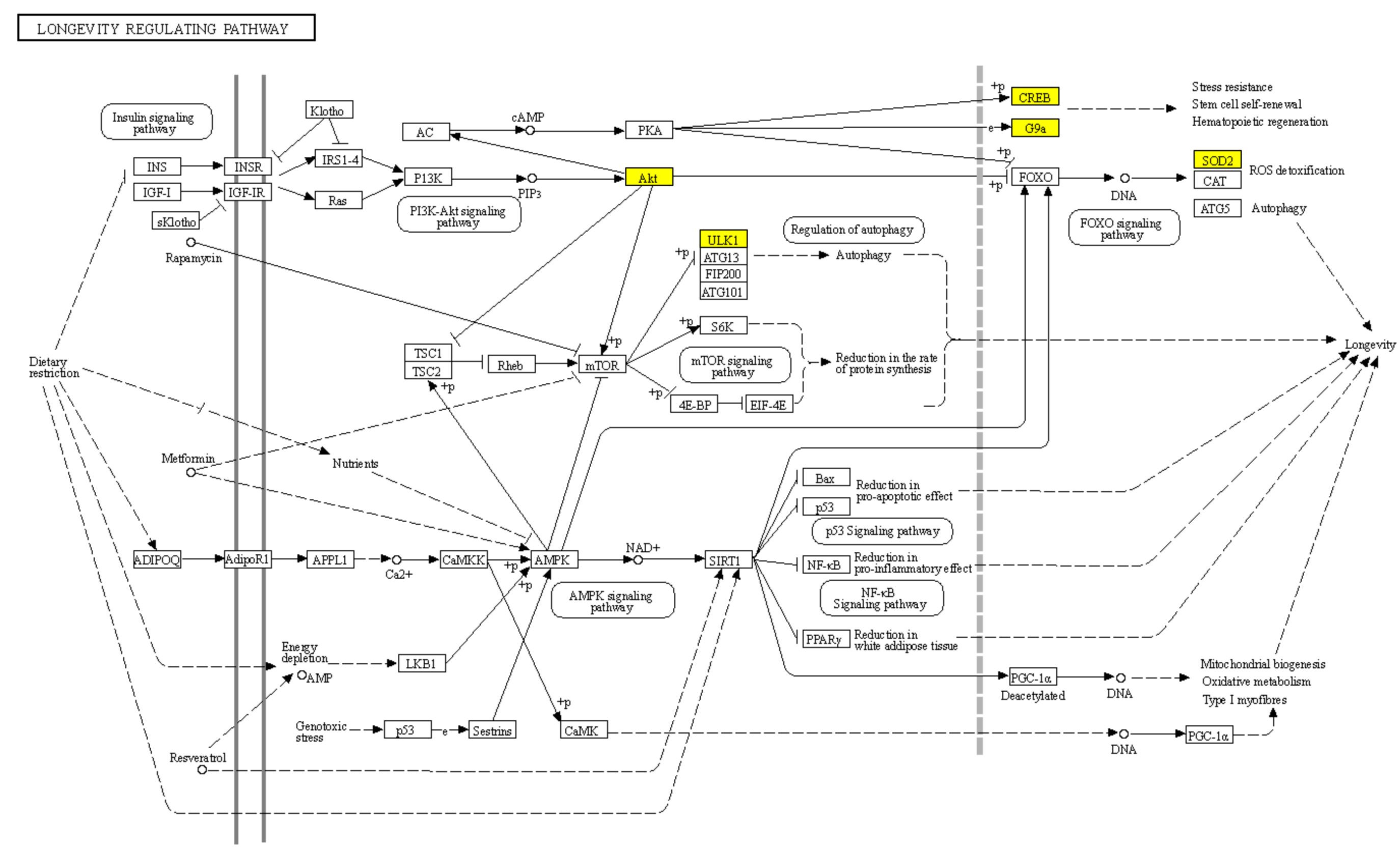


**Supplementary Figure 6:** Mapping of the longevity-associated DEGs of CUD with high anhedonia, highlighted in yellow color, on the KEGG Longevity regulating pathway.
